# Supplementary material for: Complex‐centric proteome profiling by SEC‐SWATH‐MS
Source: Mol Syst Biol. 2019 Jan 14;15(1):e8438. doi: 10.15252/msb.20188438 (PMC6346213; doi:10.15252/msb.20188438)
Supplement: Supplementary file 8 — Dataset EV7 [file MSB-15-e8438-s008.zip › feature_plots_string/Q15050.pdf]

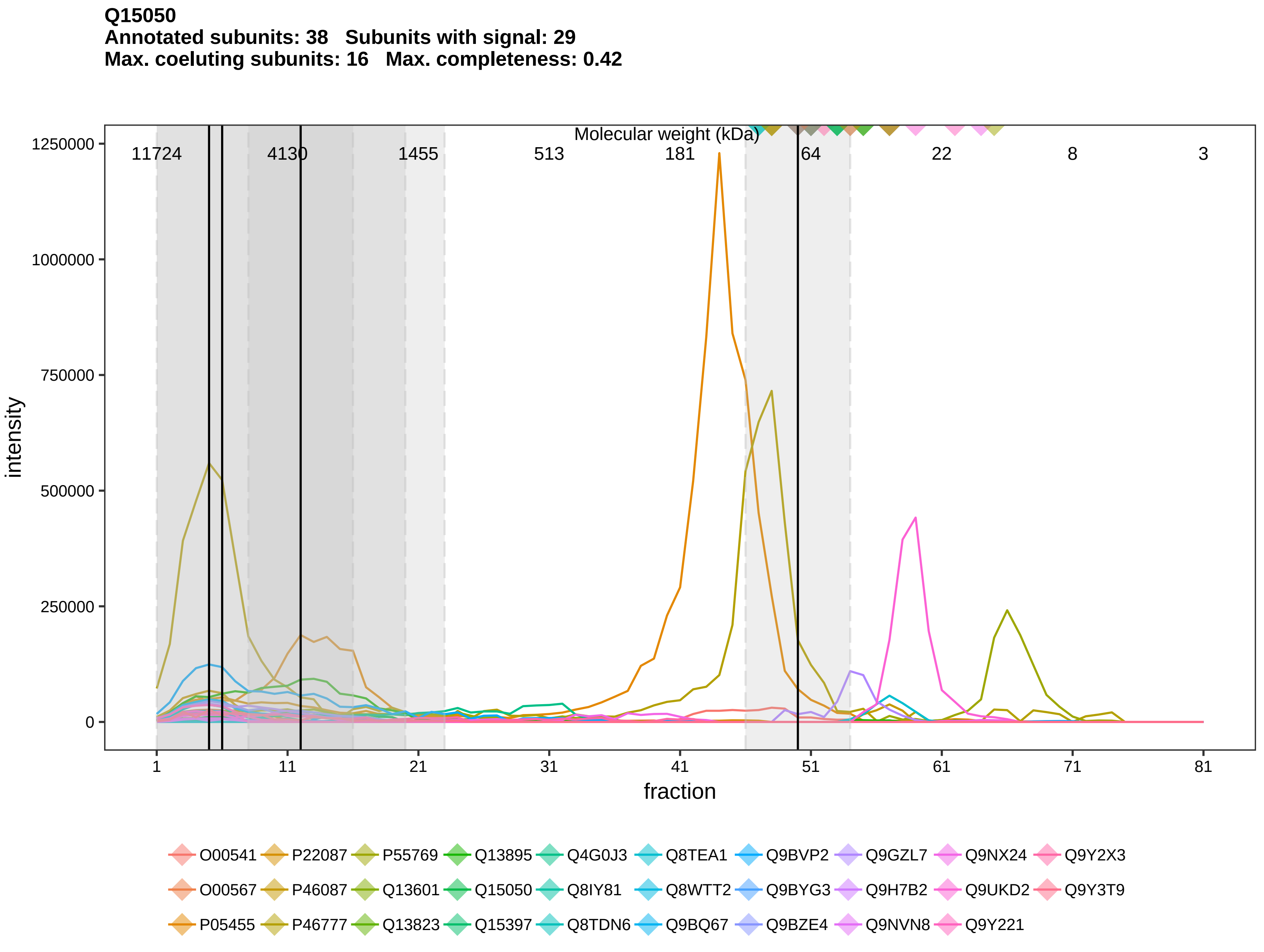
Q15050
Annotated subunits: 38 Subunits with signal: 29
Max. coeluting subunits: 16 Max. completeness: 0.42
Molecular weight (kDa)
11724
4130
1455
513
181
64
22
8
3
1250000
1000000
750000
500000
250000
0
intensity
1
11
21
31
41
51
61
71
81
fraction
O00541
P22087
P55769
Q13895
Q4G0J3
Q8TEA1
Q9BVP2
Q9GZL7
Q9NX24
Q9Y2X3
O00567
P46087
Q13601
Q15050
Q8IY81
Q8WTT2
Q9BYG3
Q9H7B2
Q9UKD2
Q9Y3T9
P05455
P46777
Q13823
Q15397
Q8TDN6
Q9BQ67
Q9BZE4
Q9NVN8
Q9Y221
